# Entabolons: How Metabolites Modify the Biochemical Function of Proteins and Cause the Correlated Behavior of Proteins in Pathways

**DOI:** 10.1021/acs.jcim.5c00462

**Published:** 2025-05-16

**Authors:** Jeffrey Skolnick, Bharath Srinivasan, Samuel Skolnick, Brice Edelman, Hongyi Zhou

**Affiliations:** † Center for the Study of Systems Biology 1372 Georgia Institute of Technology 950 Atlantic Dr NW Atlanta, Georgia 30332, United States; ‡ School of Pharmacy and Life Sciences 1018 Robert Gordon University, Aberdeen, Scotland AB10 7AQ, United Kingdom; § Cancer Research Horizons Cancer Research U.K., London CB22 3AT, United Kingdom

## Abstract

Although there are over 100,000 distinct human metabolites, their biological significance is often not fully appreciated. Metabolites can reshape the protein pockets to which they bind by COLIG formation, thereby influencing enzyme kinetics and altering the monomer–multimer equilibrium in protein complexes. Binding a common metabolite to a set of protein monomers or multimers results in metabolic entanglements that couple the conformational states and functions of nonhomologous, nonphysically interacting proteins that bind the same metabolite. These shared metabolites might provide the collective behavior responsible for protein pathway formation. Proteins whose binding and functional behavior is modified by a set of metabolites are termed an “entabolon”—a portmanteau of metabolic entanglement and metabolon. 55%–60% (22%–24%) of pairs of nonenzymatic proteins that likely bind the same metabolite have a *p*-value that they are in the same pathway, which is <0.05 (0.0005). Interestingly, the most populated pairs of proteins common to multiple pathways bind ancient metabolites. Similarly, we suggest how metabolites can possibly activate, terminate, or preclude transcription and other nucleic acid functions and may facilitate or inhibit the binding of nucleic acids to proteins, thereby influencing transcription and translation processes. Consequently, metabolites likely play a critical role in the organization and function of biological systems.

## Graphical abstract

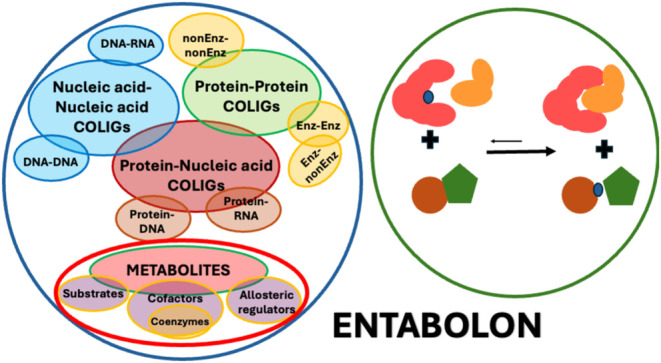

## Introduction

The interior of a cell is a remarkably crowded environment awash with many different types of molecules. For example, the molar concentration of proteins in a HeLa cell is ∼1 mM,^1^ while the total cellular concentration of free metabolites is 200 mM–300 mM;^2^ at least 31 metabolites have a concentration above 1 mM.^3^ Thus, unlike the situation for human-designed drugs where nM activities are desired,^4^ there is an excess of many metabolites relative to the total number of cellular proteins. Moreover, the human body likely contains over 100,000 distinct metabolites.^5^ Given the energy requirements to maintain such a molecular inventory, their presence seems to be a necessary component of a living system. Indeed, metabolites play important roles in transcription,^6^−,^7,8^ cellular signaling including mitochondrial nuclear communication,^9^ epigenetic regulation, phospholipid homeostasis,^10^ regulation of the immune response,^11^ and enzymatic activity.^12^ Furthermore, metabolite dysregulation is a biomarker of many diseases.^13,14^ Thus, metabolites are not passive but actively participate in many aspects of cellular function.

The recognition of the importance of metabolites in living systems spawned metabolomics, which evolved from compiling an inventory of metabolites accompanied by a purely phenomenological description of their behavior^5^ to elucidating the mechanisms by which they accomplish their biochemical and ultimately phenotypical function.^7,15^ Yet, many questions remain,^12,16^ and a general mechanistic molecular characterization of what they do in cells is lacking.^17^ Having such insight could improve the understanding of how biology works and ultimately lead to new diagnostic and therapeutic approaches.

Metabolites can modify a protein’s function by binding to the small molecule pockets present in most proteins.^18,19^ This could induce an allosteric transition within the protein, stabilize its conformation, or preclude other intermolecular interactions.^20^ Thus, depending on the context, metabolites could act as agonists or antagonists of a given molecule’s molecular function. By making such modifications, it could also change/modulate the behavior of pathways in which the protein participates. It is often assumed that such small molecule–protein interactions are highly specific. Taken to the extreme, a given enzyme might only bind those metabolites for which it is a catalyst, i.e., the “single small molecule ligand-single protein” model. If so, metabolite-protein binding would have few off-target interactions. However, the history of high-throughput small molecule screening in drug discovery contradicts this viewpoint.^21^ Moreover, given the sometimes high cellular concentrations of metabolites, there is likely a plethora of metabolites that bind with mM to nM affinities to a given protein.^22^ This was recently confirmed in ref 23 where a set of 23 diverse metabolites were shown to bind to a significant number of proteins in E. coli, termed here the Prot-Met data set (Table S1). For example, in E. coli, most proteins bind at least two different metabolites, with the number of distinct types of bound metabolites ranging from 1 to 10 (see Figure 1). The Prot-Met study was extended in ref 24 where they reached similar qualitative conclusions. While both works did not provide an in-depth elucidation of the organizing principles of metabolite–protein interactions, they do suggest that there are likely many different types of metabolites that interact with a given protein. This is consistent with other work, which demonstrated that ATP, the canonical source of cellular energy, is also a nonspecific binder to proteins.^25^ This result is not surprising given that the free cellular concentration of free ATP is ∼10 mM^3^! The picture that emerges is not of an empty pocket in a protein waiting for its high-affinity metabolite to bind; rather, most pockets are likely occupied at some point by different metabolites. Since a given metabolite likely binds a plethora of proteins, could metabolites play a role in organizing role of protein behavior?^26^

**Figure 1:**
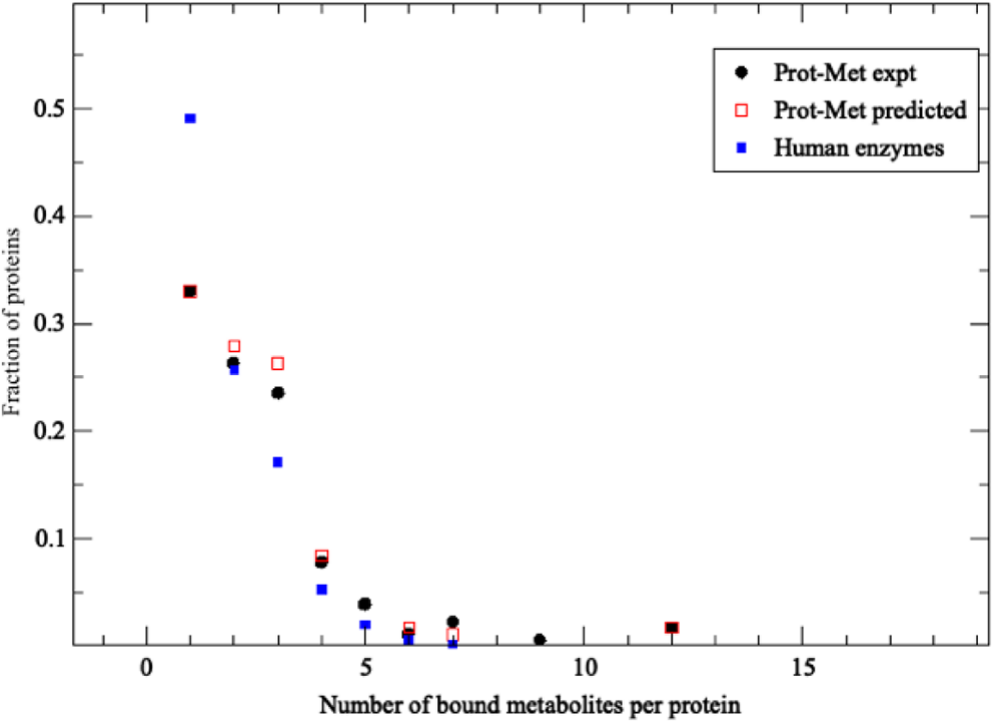
Fraction of proteins with the given number of bound types of metabolites versus the number of bound metabolites. Prot-Met data set results are in black circles, the results of LIGMAP predictions are in red squares, and LIGMAP predictions for human enzymes with PDB structures are in blue squares.

The above analysis implicitly assumed that a small molecule ligand binding pocket only binds one ligand at a time. Actually, a study of a representative set of holo (ligand-bound) Protein Databank (PDB)^27^ structures revealed that more than 50% of proteins simultaneously bind multiple interacting ligands in the same pocket.^28,29^ These interacting ligands, termed COLIGs, occupy 30%–40% of the pocket’s volume. However, the role of COLIGs in protein function was not considered; rather, this was just a structural analysis. Considering that there is a significant pool of free metabolites in a cell, for an enzyme, the binding of the second metabolite might remodel its pocket and could increase or decrease the reactant’s or product’s binding affinity. Thus, such COLIGS could directly modify the biochemical function of the given protein, and thereby they could play a role in either generating or modifying the collective behavior of proteins in pathways. Additionally, metabolite binding might induce an allosteric transition that either enhances or interferes with signaling pathways. Recently, it has been conjectured that metabolites actually are the drivers of cellular processes rather than being the passive participant in these processes.^30^ If so, the key question is how do they do this?

Life is not a noninteracting collection of molecules but depends on their collective behavior, which are often classified into pathways. The organizing principles responsible for this collective behavior are largely unknown. Clues to how the metabolites might generate such collective behavior is provided by recent studies.^31,32^ It is well known that metabolites can shift the monomer–dimer equilibrium in a set of proteins.^20,33^ Questions remain as to how this occurs. Could one predict the specific mechanism(s) underlying this equilibria? When two molecules dimerize, the region adjacent to the protein–protein interface forms a small molecule pocket similar to those in single-domain proteins.^3^ Mutations in these interface adjacent, “adj” pockets, are the second most likely cause of disease-causing variations.^31^ For 56% of protein–protein interfaces, there are also pockets in each protein’s half-interface. Another 14% of interfaces have a knobs-into-hole packing where a small molecule binding pocket in one-half interface interacts with a convex region in the other protein’s half-interface.^28^ Small molecule binding can shift the equilibrium between monomers and their dimers by binding to the adj pocket, thereby favoring dimer formation or to the monomer’s interface, thereby favoring monomer formation. The metabolite–protein interactions could also couple the monomer–dimer (or multimer) equilibrium between proteins involved in different dimers. We called this “metabolic entanglement”, as a given metabolite could couple the functions of different proteins that need not directly interact.^34^ This is a generalization of the Monod–Wyman–Changeux model, which focused on the oligomerization state of a given protein multimer. Alternatively, a metabolite might modulate the molecular behavior of monomeric proteins in a given pathway by binding to their respective pockets. This could cause an allosteric transition with the monomer or preclude or enhance the binding of its substrate (as in a COLIG). Taken together, metabolite binding could generate the collective behavior phenomenologically characterized as a protein pathway. Similarly, by attaching to the nucleic acid binding region of a protein, metabolites might prevent transcription,^35^ while those binding adjacent to the nucleic acid–protein interface might stop or initiate transcription.^36^

This paper addresses and amplifies possible roles metabolites play in modulating protein biochemical and pathway function as well as protein-nucleic acid interactions. A crucial prerequisite is the ability to predict metabolite-protein binding on a proteomic scale. Thus, we first validate a new ligand-pocket matching algorithm, LIGMAP designed to achieve this objective and examine how well LIGMAP performs in predicting known metabolite-protein pairs in enzymes. Next, for a set of 1,112 enzymes, we evaluate LIGMAP’s recall and the root-mean-square deviation, the RMSD of the predicted ligand’s binding pose when the closest template protein has <30% sequence identity to the target protein of interest. Then, we examine how COLIGs can remodel enzyme pockets and modify enzymatic interactions. COLIGs can also literally pin the native ligand to the protein. Taken together, these results show how COLIGS can modify the molecular function of individual proteins, and thereby influence the behavior of these proteins in their associated pathways. We next demonstrate that enzymes in the same pathway are coupled by binding the same COLIG ligands. Turning from the binding of monomers to dimeric proteins, we then validate the idea of “metabolic entanglement”. The results of this analysis suggest the existence of “Entabolons” defined as collections of proteins whose functional behavior is modified by a given metabolite. They may consist of monomers whose individual binding pockets are modified by metabolites, as well as monomers and multimers whose functions are coordinated by a common set of binding metabolites. Thus, the Entabolon concept generalizes the ideas of Monod, Wyman, and Changeaux.^20^ We then show that the binding of metabolites to both enzymes and nonenzymes could help form pathways. The exact molecular mechanisms by which this occurs for a particular pathway may involve the creation and collective modulation of individual protein molecular functions by COLIG formation and/or metabolic entanglement. Finally, we explore whether metabolites bind within or adjacent to protein–nucleic acid interfaces; if so, metabolic entanglement will play a role in transcription initiation and termination as well as in DNA and RNA polymerization. This study provides an additional mechanistic underpinning as to how metabolites might help regulate transcription. Overall, this work contributes additional mechanistic understanding of the coupling of metabolomics, proteomics, and transcriptomics and suggests new organizing principles of the biochemistry of life.

## Results

### Benchmarking of LIGMAP on the Prot-Met Data Set

Is it really true that proteins bind just one ligand at a time? The red squares in Figure 1, which shows the fraction of the 612 E. coli proteins in the Prot-Met data set that bind the given number of distinct metabolites, strongly argues against this idea. Almost 70% of the considered E. coli proteins bind more than one metabolite type, a result consistent with PDB structures. For protein-metabolite complexes in the PDB,^27^ one could argue that some ligands are needed to crystallize the protein, but Prot-Met and human metabolite screening^37^ involve *in vivo* data of proteins and metabolites not involved in protein crystallization. Thus, the “single small molecule ligand-single protein” model is likely an unrealistic picture for a cell.

To perform virtual ligand screening of metabolites, we developed LIGMAP. Clearly, the precision and recall of LIGMAP’s ability to predict binding ligands need to be established prior to its application to enzyme remodeling, pathway analysis, and modulation of protein–DNA binding. Here, we emphasize precision at the expense of recall as it is important to minimize the number of false positives. The precision-recall and the precision-NPV (negative predictive value) curves are shown in Figure 2 along with the corresponding random curve.

**Figure 2:**
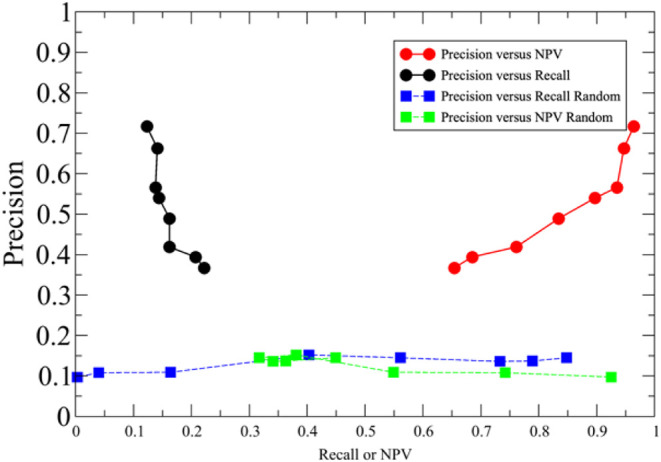
LIGMAP’s results are shown in the colored circles. The precision versus recall curves are in black, and the precision versus NPV value (defined in Table 1) are in red. The random results are shown in the squares. The precision versus recall curves are in blue and the precision versus NPV value (defined in Table 1) are in green.

LIGMAP’s results are clearly significantly better than random. Based on the performance of the precision-versus-recall curve shown in Figure 2, we opted to employ the screening criteria where the average positive predictive value (precision) (PPV) is 0.66 and the recall is 0.142.

Results for E. coli^37^ for 10 of the 20 metabolites in the Prot-Met data set, which have LIGMAP results in Prot-Met not involved in LIGMAP training (Table S1. LIST.pdb_protmet_unique), are presented in Table 1. This is a hard case as the maximum sequence identity between the template protein, which contains the bound metabolite and the corresponding target E. coli protein, is <30%. The predicted poses of the bound metabolites are found in Supporting Information (protmet_pdb).

**Table 1:** Performance of LIGMAP on the Prot-Met Dataset

Metabolite	PPV^a^	Recall^b^	NPV^c^
ADP	0.64	0.23	0.86
AKG	1.00	0.13	0.98
ATP	0.99	0.23	0.90
CIT	0	0	0.88
CMP	1	0.33	0.99
GLU	0.20	0.02	0.97
GTP	0.53	0.17	0.96
MET	0.6	0.14	0.99
PEP	1	0.13	1.00
PYR	0.67	0.03	0.95
Average	0.66	0.14	0.95

a PPV = true positives/(true positives + false positives); positive predictive value.

b Recall = true positives/(all positives).

c NPV = true negatives/(true negatives + false negatives); negative predictive value.

High-precision regime where at least 4 different templates with common ligands must be selected for the predicted target E. coli pocket.

### Screening a Representative Enzyme Library against Human Metabolites

Having established the precision of LIGMAP under the most stringent conditions of minimal sequence identity between the target and template proteins, we screened our metabolite library against enzymes. 29,581 enzymes (Table S2.LIST.fullset) were screened with the 20 metabolites from the Prot-Met set (Table S3.LIST.protmet_MET). As shown in the black circles of Figure 1, these proteins are predicted to bind at least one human metabolite in their PDB structure (SI, LIST.fullset_met). The black circles in Figure 1 show the fraction of proteins that bind the specified number of distinct ligands in the largest protein pocket, which is most likely to be functionally important.^34^ While somewhat fewer proteins bind a single metabolite compared to Prot-Met, significant portions of the curves are very similar, providing circumstantial validity of these results.

To evaluate the performance of LIGMAP, we then focused on the set of 1,112 human enzymatic proteins whose pairwise sequence identity is <90% that bind at least one metabolite in the largest ligand-binding pocket (Table S4.LIST.enzymes_aln_MET_het). As shown in Table 2, for templates whose sequence identities to the target enzyme are ≤30%, the recall of known binding metabolites is 0.86, significantly better than the Prot-Met set. This reflects the fact that these template proteins have a higher maximal sequence identity to their target. Most importantly (see column 3 of Table 2), if a metabolite is predicted to bind, then 86% of the cases select the approximate native binding pose (column 4 of Table 2) with an average root-mean-square-deviation, RMSD, of 0.73 Å, for 54 different metabolites. For a maximal target-template sequence identity <30%, <40%, <60%, or <70% the average RMSD to the native pose is 0.73, 0.70 Å, 0.66 Å, and 0.65 Å, respectively. Note that the average recall is insensitive over this sequence cutoff range, barely increasing from 0.86 to 0.89. The number of distinct metabolite types where confident predictions can be made increases from 64 to 76. Finally, if we allow all templates but the target protein itself, then the average recall is 0.92 and the average RMSD is 0.51 Å. These results suggest that if a ligand-binding prediction is made by LIGMAP, then it is likely reliable with a rather accurate pose on average. Thus, LIGMAP can be confidently used to position metabolites in their target protein structure.

**Table 2:** LIGMAP’s Recall and RMSD of Bound Native Metabolites in Enzymes

Sequence identity threshold of template to the target enzyme	Number of proteins in PDB having the bound metabolite	Recall of bound metabolites in the native pose	Number of different types of metabolites	Average RMSD for all bound metabolites in the native cluster^a^
<30%	565	0.86	54	0.73
<40%	641	0.88	64	0.70
<60%	678	0.88	72	0.66
<70%	687	0.89	76	0.65
<100%	788	0.92	112	0.51

a The native cluster is defined by metabolites within 3 Å of the native ligand.

### Role of COLIGs in Remodeling Enzyme Pockets and Modifying Enzymatic Interactions

We next turn to the issue of COLIGS in the PDB. The first four cases of Figure 3 show examples of native structures that have remodeled pockets formed by native bound COLIGs (a pair of ligands that simultaneously bind in the same pocket and that touch); at least one is a human metabolite. Figure 3A shows a partly buried pair of ligands, GLC, alpha-d-glucopyranose (red), and ADP in a glycogen synthase.^38^ Here, ADP, is a necessary component of the chemical reaction catalyzed by this enzyme.^38^ In Figure 3B, UDP, uridine 5′-diphosphate (red), and LCN 1,5-anhydro-d-arabino-hex-1-enitol (yellow), bind in a buried pocket in sucrose synthase.^39^ This structure shows how sucrose cleavage occurs as its COLIG is formed by the products of its reaction. Figure 3C shows the converse, where for a glycogen synthase,^38^ the COLIG is associated with the favorable interaction of its two reactants involved in bond ligation. Figure 3D shows a surface COLIG in ribonucleotide reductase;^40^ again, its COLIG is conjectured to be essential for its enzymatic activity. Thus, COLIGs are a likely a necessary component of enzymatic reactions; possibly other metabolites might also bind and act to modify the enzyme’s kinetics.

**Figure 3:**
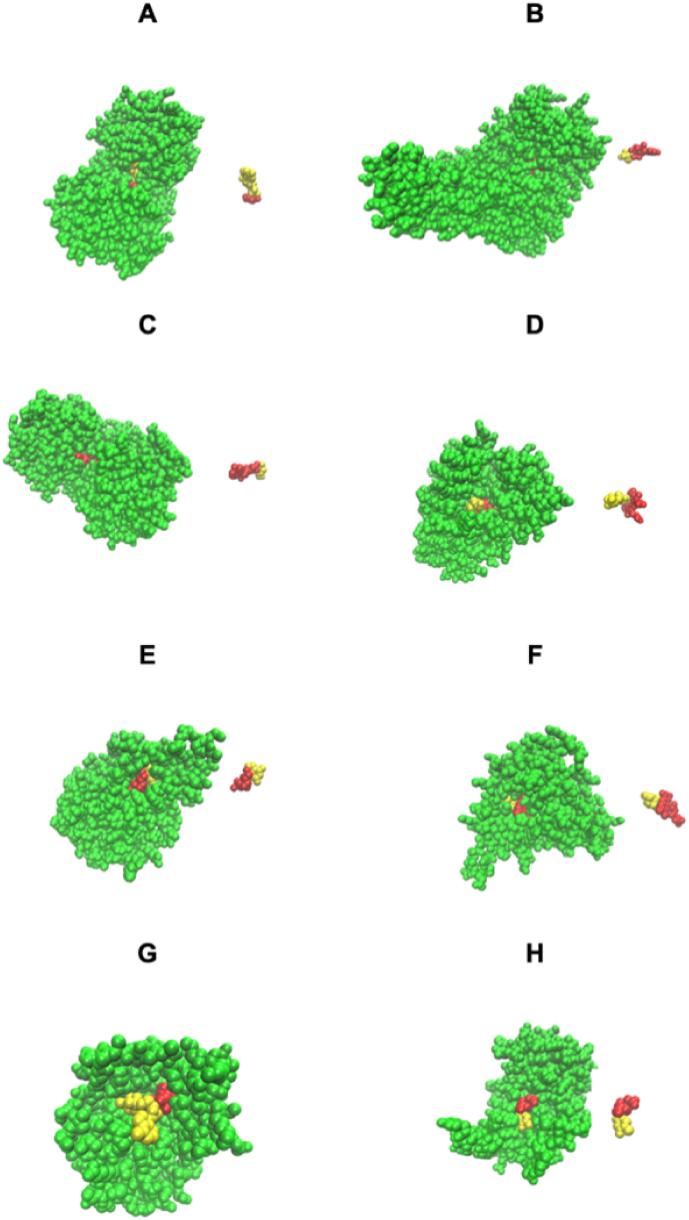
Pocket remodeling associated with native pairs of COLIGs, at least one of which is a human metabolite. The crystal structure is colored green. (A-D) Both ligands (red and yellow) are native; (E–H) the native metabolite is colored red, and the predicted COLIG metabolite is colored yellow. The right-hand structure in each panel shows the COLIGs without the protein. (A) COLIG formed by GLC (red), alpha-d-glucopyranose and ADP (yellow), and adenosine 5′ phosphate in 2r4uA,^38^ a glycogen synthase. (B) COLIG formed by enzyme reactants UDP (red), uridine 5′-diphosphate, and LCN (yellow), 1,5-anhydro-d-arabino-hex-1-enitol, buried in the interior of 3s28A, sucrose synthase-1.^39^ (C) A COLIG involved in bond ligation is shown for 3guhA, a glycogen synthase,^38^ whose COLIG is ADP, adenosine-5′-diphosphate (red), and ASO (yellow), 4-[(11beta,17beta)-17-methoxy-17-(methoxymethyl)-3-oxoestra-4,9-dien-11-yl] benzaldehyde oxime. (D) GDP (red) (guanosine 5′-diphosphate) and B12 (yellow) (cobalamin) form a surface COLIG in 3o0oA,^40^ a ribonucleotide reductase. (E) Remodeled pocket in 1a9tA,^41^ a purine nucleotide phosphorylase, whose ligands are XAN (yellow) and R1P (red), 1-*O*-phosphono-alpha-d-ribofuranose. (F) Remodeled pocket in 2dorA,^43^ a dihydroorotate dehydrogenase, whose predicted and native ligands are DOR (yellow), (4s)-2,6-dioxohexahydropyrimidine-4-carboxylic acid, and FMN (red), flavin mononucleotide. (G) Remodeled pocket in 1e2fA,^44^ a human thymidylate kinase, whose native ligand TMP (red), thymidine 5′ phosphate, has a binding surface created by ATP (yellow). (H) Remodeled pocket in 1tgyA,^27^ uridine phosphorylase, whose native ligand R1P’s (red), 1-*O*-phosphono-alpha-d-ribofuranose, binding is partly stabilized by TDR (yellow), thymine.^45^

Then, motivated by LIGMAP’s ability to quite accurately position ligands (see Table 2), we examined if LIGMAP could predict COLIGs and remodel pockets. To reduce uncertainty, we considered proteins where LIGMAP predicts a metabolite that interacts with a native bound ligand. For 440 distinct metabolites, with a quite restrictive definition of COLIGs, 12% have such COLIGs. Figure 3E shows a classic example of pocket remodeling for a purine nucleotide phosphorylase (PN),^41^ whose predicted and native ligands are XAN, xantine (yellow), and R1P, 1-*O*-phosphono-alpha-d-ribofuranose (red), respectively. Here, XAN acts to stabilize the binding of R1P to PN. XAN’s interaction with R1P to form a COLIG is plausible as PN can convert purine nucleosides into their respective bases.^42^ Figure 3F shows a significantly remodeled pocket in a dihydroorotate dehydrogenase,^43^ whose predicted and native ligands are DOR, (4s)-2,6-dioxohexahydropyrimidine-4-carboxylic acid (yellow), and FMN (red); DOR binds to other dihydrorotate dehydrogenases.^27^ Figure 3G shows pocket remodeling in a thymidylate kinase^44^ whose native ligand’s TMP, thymidine phosphate (red), binding to the protein is predicted to be enhanced by ATP. We note that ATP is known to bind this protein and is essential for its catalytic activity.^44^ Here, it is also predicted to engage in pocket remodeling to accomplish this. Finally, Figure 3H shows E. coli uridine phosphorylase,^27^ whose native ligand R1P, 1-*O*-phosphono-alpha-d-ribofuranose (red), binding is augmented by binding TDR, thymine (yellow). Interestingly, TDR is cleaved by uridine phosphorylase;^45^ an interesting prediction in that our metabolite template library does not contain TDR bound to any uridine phosphorylase. Perhaps, TDR might hold the native substrate in place prior to its reaction

Figure 3 focused on the geometric aspects of COLIG formation but did not consider whether the predicted interactions make sense from a stereochemical point of view. Figure 4 shows the same proteins as in Figure 3 except that now the types of interactions are displayed. As can be seen in Figure 4E–G, LIGMAP does a rather good job of favorably positioning the predicted COLIG ligands’ heavy atoms.

**Figure 4:**
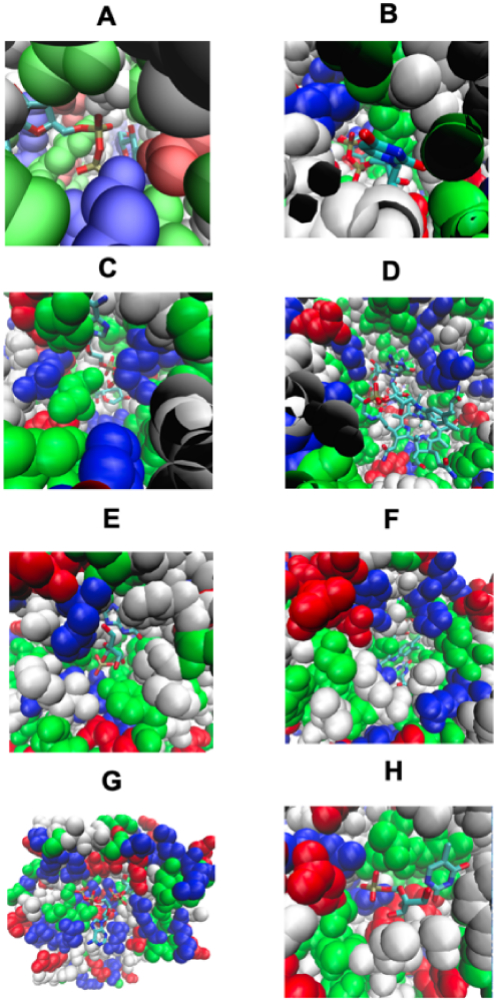
Pocket remodeling associated with native pairs of COLIGs, at least one of which is a human metabolite. Nonpolar residues are white, basic residues are blue, acid residues are red, and polar residues are green. The ligands are shown in a licorice representation, colored by the atom type with oxygen in red, nitrogen in blue, carbon in cyan, and hydrogen in white. In Figures 4A–D, both ligands are native; in Figures 4E–H, native and predicted ligands are shown. (A) COLIG formed by GLC, alpha-d-glucopyranose and ADP, adenosine 5′ phosphate in 2r4uA,^38^ a glycogen synthase. (B) COLIG formed by enzyme reactants UDP, uridine 5′-diphosphate, and LCN, 1,5-anhydro-d-arabino-hex-1-enitol, buried in the interior of 3s28A, sucrose synthase-1.^39^ (C) A COLIG involved in bond ligation is shown for 3guhA, a glycogen synthase,^38^ whose COLIG is ADP (adenosine-5′-diphosphate) and ASO (4-[(11beta,17beta)-17-methoxy-17-(methoxymethyl)-3-oxoestra-4,9-dien-11-yl] benzaldehyde oxime). (D) GDP (guanosine 5′-diphosphate) and B12 (cobalamin) form a surface COLIG in 3o0oA,^40^ a ribonucleotide reductase. (E) Remodeled pocket in 1a9tA,^41^ a purine nucleotide phosphorylase, whose ligands are XAN and R1P (1-*O*-phosphono-alpha-d-ribofuranose). (F) Remodeled pocket in 2dorA,^43^ a dihydroorotate dehydrogenase, whose predicted and native ligands are DOR ((4s)-2,6-dioxohexahydropyrimidine-4-carboxylic acid) and FMN (flavin mononucleotide). (G) Remodeled pocket in 1e2fA,^44^ a human thymidylate kinase, whose native ligand TMP, thymidine 5′ phosphate, has a binding surface created by ATP. (H) Remodeled pocket in 1tgyA,^27^ uridine phosphorylase, whose native ligand R1P’s, 1-*O*-phosphono-alpha-D-ribofuranose, binding is partly stabilized by TDR, thymine.^45^ The figures were generated by VMD.^46^

### Ligand Pinning COLIGs

Turning to the case where one ligand pins the other to the protein and blocks its release, we first consider native COLIGs. Figure 5A shows a particularly salient case where native HSO (red), l-histidinol, is totally pinned in its pocket by HEM (yellow) in a peroxidase.^47^ Here, the protein’s pocket is deep inside the native structure. In Figure 5B, IMP, inosinic acid (red), is pinned by NAD (yellow) to the side of the protein pocket in a human inosine monophosphate dehydrogenase.^48^ Figure 5C is interesting in that its two ligands, CLA, chlorophyll A (red), and DGD, digalactosyl diacyl glycerol (yellow), are intertwined in 5h2fC.^27^ Finally, Figure 5D shows another example of complete pinning in a human dihydroorotate dehydrogenase,^49^ where FMN (red) is totally blocked by ORO, orotic acid (yellow).

**Figure 5:**
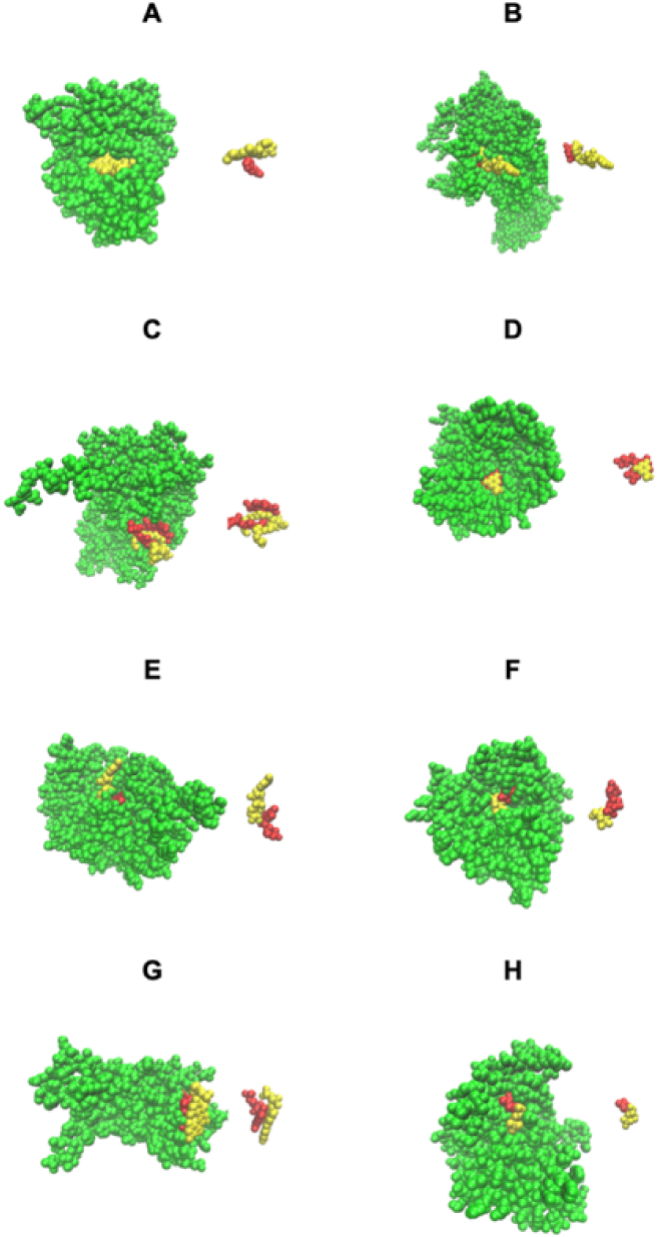
Examples of ligand pinning COLIGs in native PDB structures. The native protein structure is colored green. (A–D) Both ligands (red and yellow) are native; (E–H) the native metabolite is colored red, and the predicted metabolite is colored yellow. The right-hand structures in each panel show the COLIGS with their native structure removed. (A) HEM (yellow), protoporphyrin IX pins HSO (red), l-histidinol in1h3jA,^47^ a peroxidase. (B) NAD (yellow), nicotinamide-adenine-dinucleotide, pins IMP, inosinic acid (red) in 6ua2A, human inosine monophosphate dehydrogenase.^48^ (C) Example of intertwined pocket remodeling/ligand pinning, by CLA, chlorophyll A (red), and DDG, digalactosyl diacyl glycerol (yellow) in 5h2fC,^27^ part of photosystem II. (D) FMN, flavin mononucleotide (red), is totally pinned by ORO (yellow), orotic acid in 6fmdA, human dihydroorotate dehydrogenase.^49^ (E) Native ligand FAD (red) forms a COLIG with MLI (yellow), malonate ion that partially blocks its exit from 1bwkA,^50^ old yellow enzyme. (F) The native ligand, UDP (red), uridine-5′-diphosphate, is partially pinned by GLC (yellow), alpha-d-glucopyranose in1k4vA,^51^ beta-galactoside-alpha-1,3-galactosyltransfera. (G) The native ligand, FMN (red), flavin adenine dinucleotide, is pinned by FMN (yellow), flavin mononucleotides in 3r6wA,^53^ an azoreductase. (H) The native ligand TDR (red), thymine, is pinned to the side of the pocket by R1P, 1-*O*-phosphono-alpha-d-ribofuranose, in 4txm, a uridine phosphatase.^55^ The figures were generated by VMD.^45^

Predicted ligand pinning COLIGs are quite rare, occurring in ∼0.4% of screened enzymes. Figure 5E–H shows four examples where the predicted metabolite in the predicted metabolite-native COLIG pair pins the native metabolite to its native structure. In Figure 5E, for old yellow enzyme’s^50^ native ligand FAD (red), MLI (yellow), malonate ion, blocks one exit from the pocket. This is possible as the cytoplasmic concentration of MLI is 1–2 mM^3^. In Figure 5F, beta-galactoside-alpha-1,3-galactosyltransferase’s^51^ native ligand, UDP (red), is predicted to be partly pinned by GLC (yellow), alpha-d-glucopyranose; since the cellular concentration of GLC is several millimolar, this is possible.^52^ Figure 5G is interesting in that the predicted metabolite FMN (yellow) “hugs” the native ligand, FMN (red), in an azoreductase enzyme.^53^ Note that the free concentration of FMN in cells is quite low. Interestingly, the second FMN binds to the protein in addition to the other FMN molecule; FMN forms dimers in water^54^ at 0.3 mM concentrations, so this COLIG might occur. Figure 5H shows a uridine phosphorylase, 4tmxA,^55^ that has its native ligand TDR (red), thymine, pinned to the side of the pocket by R1P (yellow), 1-*O*-phosphono-alpha-d-ribofuranose. This is very similar to the native COLIG that was shown in Figure 5D.

We next display in Figure 6 the corresponding set of stereochemical interactions that once again show that LIGMAP generates plausible detailed atomic models of ligand pinning COLIGS.

**Figure 6:**
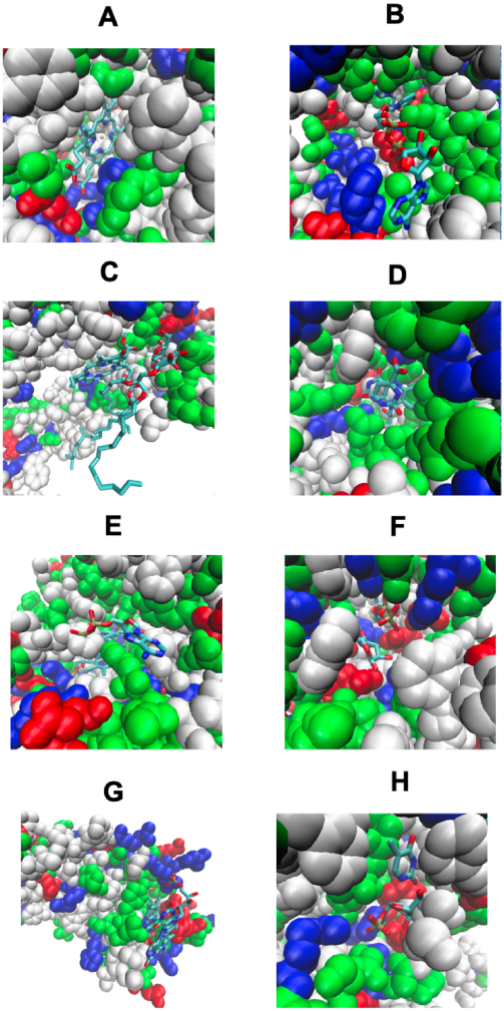
Examples of ligand pinning COLIGs in native PDB structures. Nonpolar residues are white, basic residues are blue, acid residues are red, and polar residues are green. The small molecule ligands are shown in a licorice representation, colored by atom type in the protein with oxygen in red, nitrogen in blue, carbon in cyan, and hydrogen in white. (A–D) Both ligands are native; (E–H) native and predicted metabolites are shown. (A) HEM, protoporphyrin IX, pins HSO, l-histidinol in1h3jA,^47^ a peroxidase. (B) NAD, nicotinamide-adenine-dinucleotide, pins IMP, inosinic acid in 6ua2A, human inosine monophosphate dehydrogenase.^48^ (C) Example of intertwined pocket remodeling/ligand pinning, by CLA, chlorophyll A, and DDG, digalactosyl diacyl glycerol, in 5h2fC,^27^ part of photosystem II. (D) FMN, flavin mononucleotide, is totally pinned by ORO, orotic acid, in 6fmdA, human dihydroorotate dehydrogenase.^49^ (E) Native ligand FAD forms a COLIG with MLI, malonate ion, that partially blocks its exit from 1bwkA^50^ 6, old yellow enzyme. (F) The native ligand, UDP, uridine-5′-diphosphate, is partially pinned by GLC, alpha-d-glucopyranose in1k4vA,^51^ beta-galactoside-alpha-1,3-galactosyltransfera. (G) The native ligand, FMN, flavin adenine dinucleotide, is pinned by FMN, flavin mononucleotides in 3r6wA,^53^ an azoreductase. (H) The native ligand TDR, thymine, is pinned to the side of the pocket by R1P, 1-*O*-phosphono-alpha-d-ribofuranose, in 4txm, a uridine phosphatase.^55^ The figures were generated by VMD.^45^

### Implications of COLIGS for Bisubstrate Enzymes

More generally, bisubstrate enzymes or enzymes with more than two substrates^56^ exemplify ligand-assisted protein pocket remodeling that are the result of COLIG formation. These are sequential reactions where both substrates bind the enzyme before its products are released. In random sequential reactions, substrate binding order does not matter, and COLIG formation is independent of the small molecule-protein interface created to accommodate the second ligand. However, in ordered sequential reactions, the order of substrate binding is important and are prototypical examples of how a COLIG is defined. Almost all enzymes operate with either two or a greater number of substrates, and COLIG based chemistry optimization is the only way to organize metabolic cascades. Finally, it has not escaped our notice that the conventional definition of a molecular glue^57^ is actually a COLIG. In a molecular glue, the binding of the small molecule glue changes the molecular interaction surface thereby facilitating novel interactions. Molecular glues have had a tremendous reception in the pharmaceutical community and show immense promise for treating rare disorders unaddressed by traditional approaches. We posit that metabolism has harnessed the power and modularity of naturally occurring molecular glues within the framework of COLIGs to facilitate and organize macromolecular clustering and pathway creation.

### Enzymes in the Same Pathway are Coupled by Binding the Same COLIG Ligands

A representative set of 2233 enzymes was identified that had predicted COLIGS (Table S5.LIST.enzymes_colig). These proteins can be assigned to 376 distinct pathways by the REACTOME pathway knowledgebase^58^ or KEGG.^59^ We then explored what fraction of the enzymes belonging to the same pathway could be connected by COLIGS. In a given pathway, a set of proteins is defined as being COLIG-connected when all of these proteins bind the same COLIG metabolite. A protein that binds two or more metabolites acts as a junction that allows the union of the two COLIG-connected sets of proteins. Remarkably, 91% of the proteins in these pathways have assigned COLIGS that are coupled by binding an overlapping set of metabolites (see SI, stat_colig_pathway). This suggests that metabolites could be at least partially responsible for the generation of the cooperative protein behavior that characterizes a protein pathway. This idea is explored in greater detail in what follows.

### Evidence for the Metabolic Entanglement of Protein Pathways

On binding to a given protein, the given metabolite stabilizes that structure. In Figure 7, we expand upon this fact and consider a subset of the elementary situations (and their associated equilibria) that shows how metabolite binding can perturb monomer–dimer equilibria. It can also act to couple the state of association of sets of *noninteracting proteins*. Figure 7A shows the simplest, well-known situation where the metabolite stabilizes the dimer relative to its monomers. Now we just show one mechanism by which this can be achieved. When the metabolite dissociates from the dimer, the dimer also dissociates into its monomers. If the dimer contains the requisite function for the given pathway, metabolite dissocation eliminates this function. However, if the function resides in the monomer, then metabolite dissociation from the dimer would enable its function. In Figure 7B, metabolite binding to the interface-adjacent region induces an allosteric transition in the dimer. Perhaps one of these two states (metabolite-bound or unbound) is the functionally competent state for the pathway (the same ideas hold for higher-order multimers). Similarly, metabolite binding to a given monomer (not shown) could result in an allosteric transition that allows or abolishes the protein’s function. These suggestions are concrete realizations of the Monod–Wyman–Changeux models.

**Figure 7:**
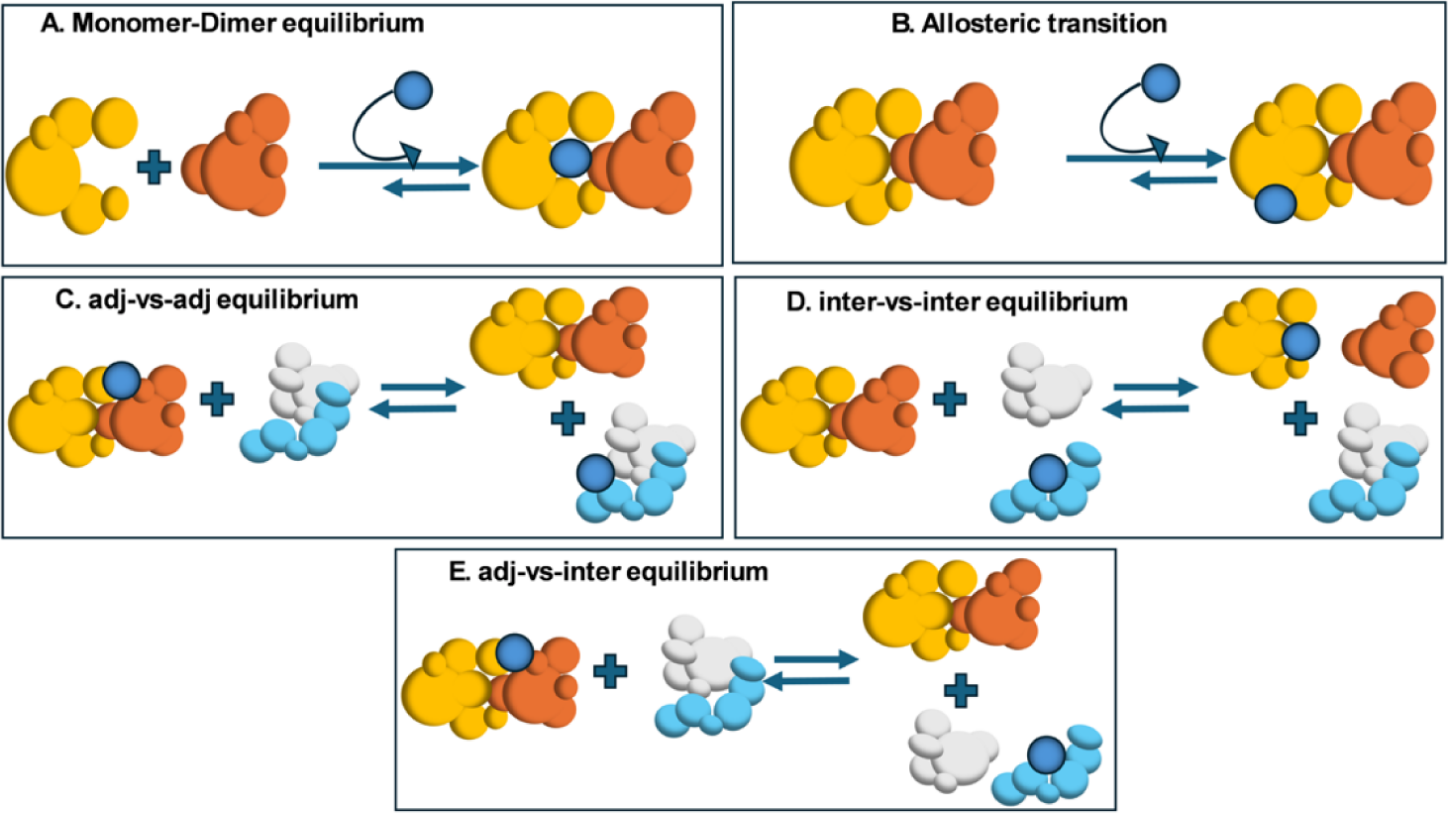
Examples of “metabolic entanglement.” (A) Metabolite (dark blue) binding to the pocket adjacent to the protein–protein interfaces stabilizes the dimer relative to its dissociated monomers. This shifts the monomer–dimer equilibrium. (B) Binding of the metabolite (dark blue) induces an allosteric transition in the dimer. (C) The “adj-vs-adj” case considers situations where the metabolite (dark blue) binds adjacent to the protein–protein interface in two distinct dimers. Both molecules remain in the dimeric state, but the presence and absence metabolite binding induces an allosteric transition within the pair of dimers. (D) The “inter-vs-inter” case occurs when the binding of a metabolite (dark blue) to an interfacial pocket in a monomer precludes dimer formation. Dissociation of the metabolite from the light blue monomer allows it to bind to an interfacial pocket in another monomeric (orange) protein. This induces dissociation of the left-hand (yellow/orange) dimer and association of the light blue and white monomers to form a dimer. (E) The “adj-vs-inter” case involves the shift of equilibrium of the yellow/orange dimer containing the interface adjacent metabolite (dark blue) to the interfacial pocket in one of the chains of the light blue/white dimer. This dissociates the light blue/white dimer and might cause an allosteric transition within the yellow/orange dimer.

The aforementioned cases are not novel. We now consider new situations. Previously,^34^ we conjectured the existence of “metabolic entanglement,” which refers to the shift in conformational equilibrium between pairs of protein that need not directly interact but whose conformational state is modified by the presence or absence of a common binding metabolite. This differs from the idea of a metabolon, which involves a temporary complex of enzymes in a cell that help to channel the metabolic reactants and protein through a pathway.^60^ Here, the proteins that bind the metabolite need not be enzymes and do not directly interact. Different possible elemental equilibria are shown in Figure 7C–E. In Figure 7C, the metabolite binds to the region adjacent to the protein–protein interface in one of the two distinct dimers, the “adj-vs-adj” equilibrium. When the metabolite is bound to the other dimer because its free energy is lower, this not only increases the stability of that dimer but might induce allosteric transitions within both dimers, possibly modifying their biochemical functions. Note that the pair of dimers do not directly interact with each other. Figure 7D depicts the “inter–inter” equilibrium where the dissociation of the metabolite bound to an interfacial pocket in one (light blue) of the monomers shifts the equilibrium and induces its dimerization with the white protein. Now the metabolite is no longer bound to the light blue molecule nor its partner white molecule. However, the metabolite now binds to a pocket in the interface of the yellow protein, thereby causing the yellow/orange dimer to dissociate. Thus, metabolites can turn off functions if the functional site is in either the light blue (metabolite bound) or white monomer or create new functions if the white/light blue dimer and/or the orange or yellow (metabolite bound) monomers are functional. Finally, Figure 7E considers the equilibrium shift between two dimers where the metabolite bound adjacent to its interface in the yellow/orange dimer dissociates to yield the yellow/orange dimer which may undergo an allosteric transition and two monomers (light blue and white), one of whose interfacial pocket (light blue) binds the metabolite, the “adj-vs-inter” case. Such population shifts can introduce or eliminate functional conformations.

With regards to pathways, we conjecture that metabolite entanglement might tie together the members of the pathway and generate their collective behavior. The logical implication is that metabolic entanglement might actually create as well as regulate a given pathway. Strong evidence for this conjecture is provided in Table 3 for the three types of metabolite binding. Here, we only consider pairs of proteins such that the sequence of a monomer of a given dimer protein (by itself or existing as a dimer) is no more than 40% sequence identical to any of the monomers in the other dimer. We consider cases where both protein dimers are enzymes, “Enz vs Enz”, both are nonenzymes, “Non-Enz vs Non-Enz”, and one protein dimer is enzyme and the other is a nonenzyme, “Enz vs Non-Enz”. As it might be expected, metabolic entanglement is most effective for “Enz vs Enz”. Note that fraction of pairs of proteins in the same pathway that bind the same metabolite whose *p*-value <0.05 is largest for the inter vs inter case, 0.78. Significantly, 43% of pairs have a *p*-value <0.0005. The next most significant case is “adj vs inter” where 75% (35%) of enzyme pairs have a p-value ≤0.05 (0.0005). While one might argue that this just reflects the binding of the metabolite to the component monomers, “adj vs adj” strongly argues that this is not the case; 73% of pairs have a *p*-value ≤0.05 and 33% have a p-value **≤**0.0005.

**Table 3:** *P*-Value for the Likelihood That a Pair of Proteins Binding the Same Metabolite Occurs in the Same Pathway

Type of protein pair		Fraction of pairs of proteins per metabolite averaged over metabolites whose *P*-value is ≤ the given threshold					
	No. of metabolites	No. of protein pairs in the same pathways	0.05	0.01	0.005	0.001	0.0005
adj vs adj							
Enz vs Enz^a^	132	2478	0.73	0.46	0.43	0.37	0.33
Non-Enz vs Non-Enz^b^	57	515	0.55	0.40	0.36	0.26	0.22
Enz vs Non-Enz^c^	50	878	0.60	0.40	0.35	0.23	0.20
inter vs inter							
Enz vs Enz^a^	173	4941	0.78	0.57	0.55	0.47	0.43
Non-Enz vs Non-Enz^b^	52	406	0.57	0.39	0.37	0.32	0.23
Enz vs Non-Enz^c^	67	1157	0.61	0.43	0.36	0.27	0.24
adj vs inter							
Enz vs Enz^a^	169	6841	0.75	0.50	0.47	0.40	0.35
Non-Enz vs Non-Enz^b^	65	897	0.60	0.42	0.38	0.30	0.24
Enz (adj) vs Non-Enz (inter)	102	1739	0.61	0.39	0.35	0.26	0.23
Non-Enz (adj) vs Enz (inter)	86	2550	0.61	0.44	0.39	0.26	0.23

a Both proteins are enzymes.

b Both proteins are nonenzymes.

c One protein is an enzyme and the other is a nonenzyme.

While the “Enz vs Enz” provides strong circumstantial evidence for the existence of metabolic entanglement, one could also argue that since enzymes are involved in the production and degradation of metabolites, all one is exploring is standard effects of metabolic flux within an enzymatic pathway.^59^ This viewpoint is refuted by the “Non-Enz vs Non-Enz” results. The “adj vs adj”, “inter vs inter” and “adj vs inter” cases have roughly 55%, 57% and 60% of pairs having a p-value ≤0.05 that they occur in the same pathway, and 22%, 23% and 24% of protein pairs respectively have a p-value ≤0.0005! Here the adj vs inter pairs are most significant. Thus, metabolites couple the presence of pairs of nonenzymes in the same pathway, with similar results found for the Enz vs Non-Enz cases.

Just as COLIGS within a given pathway are predicted to bind to most of the proteins found in the same pathway, metabolically entangled proteins cover most proteins involved in quite complex pathways. On average 78% of the proteins in 69 distinct pathways involving the proteins analyzed above are metabolically entangled, with the top 15 pathways shown in Table 4. Table 4 also provides a list of the entangling metabolites, ranked by the number of pairs of proteins within a given pathway that they bind to. All that is required to achieve these results is that a given dimer or its composite monomers bind at least two metabolites. In reality, since we only consider 770 metabolites and use LIGMAP whose recall is not 100%, in cells the effects of metabolic entanglement would be even larger. Thus, metabolic entanglement is operative in a diverse collection of pathways including those involved in the immune system, cholesterol biosynthesis, the cell cycle and cellular senescence, as well as in cancers such as glioma.

**Table 4:** Metabolic Entanglement in Representative Pathways

Reactome Pathway	Number of proteins binding the entangling metabolites	Number of proteins in the given pathway^a^	Ratio	Metabolites (LLM supported) (LLM mixed)^a^ ^,^ ^b^				
Immune system	49	53	0.925	GOL	PO4	ACT	FLC	CIT
Regulation of cholesterol biosynthesis by SREGP	34	38	0.895	GOL	ACT	PO4	OLA	MYR
MAPK signaling pathway	21	24	0.875	GOL	ACT	PO4	TMO	
UCH proteinases	21	24	0.875	GOL	PO4	ACT	CIT	TMO
Ubiquitin-dependent degradation of cyclin D	20	23	0.87	GOL	PO4	ACT	CIT	TMO
Toll Like Receptor 5 (TLR5) cascade	20	23	0.87	GOL	PO4	ACT	ACY	D12
RHO GTPases activate ROCKs	20	22	0.909	GOL	CIT	ATP	FMN	GDP
Cell cycle—G1/S transition	15	17	0.882	ACT	GOL	PO4	ADP	CIT
″Cell cycle mitotic″	14	16	0.875	ACT	GOL	PO4	AMP	ATP
Cellular responses to stress	13	15	0.867	GOL	ACT	PO4	ATP	CIT
Cellular senescence	12	14	0.857	ACT	GOL	PO4	ATP	ADP
Chronic myeloid leukemia	12	14	0.857	ACT	ADP	AMP	ATP	GOL
Cyclin D associated events in G1	12	14	0.857	ACT	GOL	AMP	ATP	ADP
G1 phase	11	13	0.846	GOL	ACT	PO4	ATP	ADP
Glioma	11	13	0.846	GOL	ACT	AMP	ATP	ADP

a The LLM-based literature support provided by ValSci.

b GOL is glycerol, CIT is citric acid, FLC is citrate, MYR is myristic acid, ACT is acetate, TMO is trimethylamine oxide, and D12 is dodecane.

We briefly describe evidence supporting these predictions below. The MAP (mitogen-activated protein) kinase pathway is a critical signaling cascade that regulates various cellular processes such as growth,^61^ differentiation,^62^ and stress responses.^63^ It involves a series of phosphorylation events, where MAP kinases (such as ERK,^64^ JNK,^65^ and p38 ^66^ are activated by upstream kinases, ultimately leading to changes in gene expression and cellular behavior. The pathway is triggered by various receptors, including receptor tyrosine kinases (RTKs), which activate small GTPases like Ras.^67^ These molecules activate a kinase cascade, involving MAPKKs (MAP kinase kinases) that phosphorylate and activate MAPKs. All of the predicted metabolites for the MAP pathway shown in Table 4 have experimental support. The paper by Torres-Quiroz et al.^68^ demonstrates that the HOG MAPK pathway is involved in glycerol accumulation during endoplasmic reticulum (ER) stress. The study shows that Hog1p, a MAPK, regulates the transcription of GPD1, a gene involved in glycerol synthesis, and that glycerol accumulation is dependent on Hog1p activity. In primary astrocytes, acetate treatment was found to decrease the basal levels of phosphorylated ERK1/2 by 2-fold and completely reversed LPS-induced phosphorylation of MAPK p38.^69^ Furthermore, changes in PO4 levels may alter MAPK activity both directly (by impacting the phosphorylation process) and indirectly through cellular responses to phosphate stress.^70^ Trimethyl amine oxide (TMO) is known to activate the MAPK signaling pathway.^71^ Thus, these metabolites provide the cell with a way to integrate environmental and metabolic cues into the signaling processes that govern cell fate decisions. Metabolic entanglement-mediated organization of this pathway’s players (Table 4) emphasizes the importance of our study in shedding further light on how metabolites are pivotal in organizing this critical pathway with an important role in oncogenesis.

Turning to the other pathways of Table 4, there is some evidence that citric acid and citrate, CIT and FLC, may play an important role in immunity and inflammation.^72^ MYR, myristic acid is known to raise cholesterol levels, in particular LDL levels.^73^ AMP, ADP^74^ and ATP^75^ play an important role in glioma. To further assess the possible accuracy of predicted metabolites and perform a complementary analysis, we used Valsci, an open-source literature review tool. ValSci integrates gpt-4o large language models with chain-of-thought reasoning to determine the relevance, support, and contradicting evidence found in the Semantic Scholar^76^ database of over 80 million academic papers and abstracts, with the reports provided in Table S4_valsci. Green indicates that the particular predicted metabolite is strongly supported by the literature and orange is that it has mixed evidence. 36/75 predictions have literature support with the remainer lacking such support. They may be incorrect or novel but correct predictions. Thus, the evidence for metabolic entanglement being an important component of pathway regulation and possibly creation is significant.

### Binding of Metabolites to Nonenzymes Also Helps Create Pathways

We next considered a set of 4779 (Table S6.LIST.nonenzymes_human) human protein monomers that are not enzymes and examined the fraction of monomeric proteins involved in 209 pathways that bind a common set of ligands and perform the same analysis, with the results summarized in Table 5. On average, 85% of the proteins in these pathways bind a common set of metabolites. Unlike in Table 4, here we excluded glycerol from the analysis and required that each metabolite have at least four examples of stereochemically similar pockets in the template proteins. Based on Table 1, their predicted precision is 0.64. Again, listed metabolites are ranked by the number of proteins that they bind. Since just monomers and not their multimeric complexes are considered, for common pathways, the number of protein pairs that a given metabolite binds is larger and the selected top-ranked metabolites can be different. The top 24 pathways are shown in Table 5. Many have circumstantial evidence that they are at least associated with the biochemical processes associated with the given pathway. ATP, ADP,^77^ OLA,^78^ and GDP^79^ are all associated with signal transduction, Dimethyl lysine (MLY) is known to be associated with gene expression.^80^ Phosphatidyl choline (PLC) has been implicated in neuroactive ligand receptor interactions;^81^ phosphoserine PTR plays a role in cellular homeostasis.^82^ Palmitic acid PLM can significantly inhibit neutrophil degranulation,^83^ while oleic acid (OLA) is involved in cytokine signaling,^84^ colic acid (CHD) plays a key role in lipid metabolism.^85^ Finally, dimethyl lysine (MLY)^86^ and phosphoserine^87^ are cancer associated. We then applied Valsci to these results; 54/96 of the predicted metabolites have at least some literature support, see Table S5_valsci.

**Table 5:**
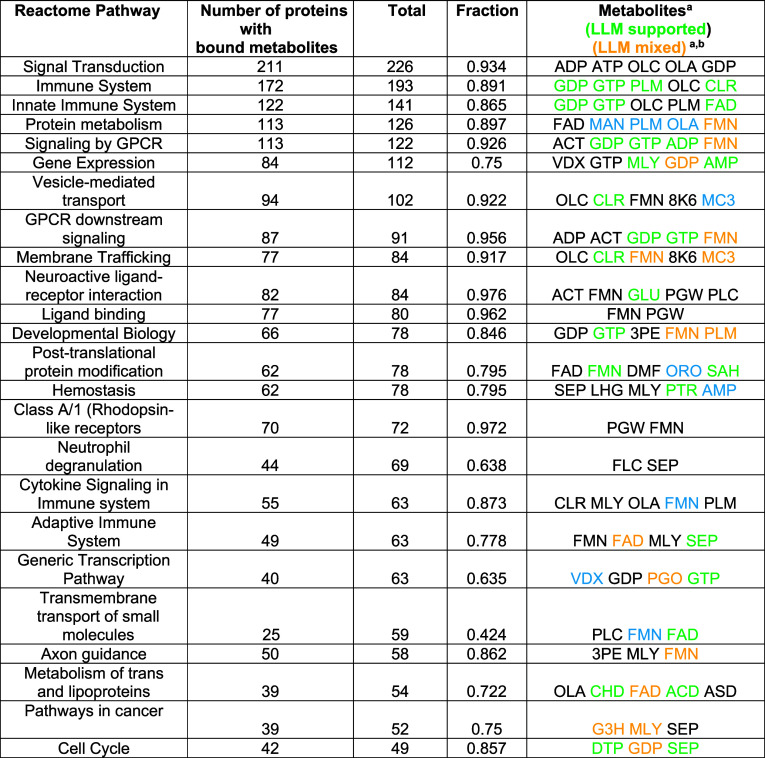
Metabolic Coupling of Monomers in Representative Pathways

a The LLM based literature support provided by Valsci.

b OLC is 2-3 dihyroxypropyl-ocadec-enoate, OLA is oleic acid, PLM is palmitic acid, CLR is cholesterol, ACT is acetate, VDX is 5-(2-[1-(5-hydroxy-1,5-dimethyl-hexyl)-7a-methyl-octahydro-inden-4-ylidene]-ethylidene)-4-methylene-cyclohexane-1,3-diol, MLY is N-dimethyl lysine, 8K6 is octadecane, MC3 is 1,2 dimyristoyl-rac-glycero-3-phosphocholine, SEP is phosphoserine, GLU is glutamic acid, PGW is (1*R*)-2-[(*S*)-{[(2*S*)-2,3-dihydroxypropyl]­oxy}­(hydroxy)­phosphoryl]­oxy)-1-[(hexadecanoyloxy)­methyl]­ethyl (9*Z*)-octadec-9-enoate, 3PE is 1,2-distearoyl-*sn*-glycerophosphoethanolamine, DMF is dimethylformamide, ORO is orotic acid, SAH is *S*-adenyosyl-l-homocysteine, LHG is 1,2 dipalmityol-phosphatidyl-*O*-glycerol, PTR is *O*-phosphotyrosine, PGO is 1,2 propane diol, CHD is cholic acid, ACD is arachidonic acid, ASD is 4-androstene-3-7-dione, and DTP is 2’-deoxtadenosine-5′-triphosphate.

We next ranked the frequency that a given metabolite binds to two or more proteins, with the full list provided in Supporting Information histogram_metabolites.txt. The top-ranked ligand is phosphoserine, which is found in all living organisms and has some evidence that it is an ancient metabolite.^88^ The next most frequent metabolite is FMN, which is also considered to be an ancient metabolite.^89^ This is followed by MLY, *N*-dimethyl lysine, whose ubiquitous presence in many organisms is also consistent with the idea that it is another ancient metabolite.^5^ The next two, GDP and GTP, are also considered ancient metabolites^90^ as is FAD.^91^ This is followed by PLM, palmitic acid, which is also found in all living organisms, suggestive that it might be an ancient metabolite.^92^ Then RET, retinal is another ancient metabolite.^93^ It is followed by OLA, oleic acid whose presence in bacterial, plant and animal membranes suggests that it is an ancient metabolite.^92^ The tenth-ranked metabolite is cholesterol, which is predominantly found in animals; the ability to produce sterols is at least a billion years old.^94^ Thus, the top 10 most prevalent metabolites are common to at least animals if not all branches of life. This is suggestive that their interactions with a set of proteins could give rise to the collective behavior of pathways and supports the idea of the existence of Entabolons.

### Roles of Metabolites in Modulating Nucleic Acid Function

As was the case with adj and inter-bound metabolites that modulate protein–protein interactions, metabolites could similarly modulate nucleic acid–protein interactions and nucleic acid–nucleic acid interactions. If they competitively bind to the DNA/RNA-protein interface, they could preclude the DNA/RNA-protein interaction from happening. What is less known is that metabolites could also act as DNA/RNA-protein binding agonists or antagonists by binding to the region adjacent to the DNA–protein interface. Likewise, an expanding repertoire of tRNA modifications has been implicated as being facilitated by metabolic signaling pathways that have yet to be fully characterized. We used LIGMAP to select metabolites that could selectively bind to the DNA–protein interfacial region. Figure 8A shows the binding of DCP, DTP, and ATP to the end of DNA which is bound to Y-family polymerase 4, 3khhA;^95^ DCP, 2’-deoxycytidine-5′-triphosphate, and DTP, 2’-deoxyadenosine-5′-diphosphate, are both involved in DNA polymerization,^99^ and ATP plays an indirect role in providing DNA building blocks. We consider another DNA polymerase in Figure 8B whose large fragment of Taq DNA polymerase, 3t3fA,^96^ is predicted to bind 31 small molecules including human metabolites DCP, DGT, and ACT, whose role in DNA polymerization is not established. Figure 8c shows the structure of aprataxin, 6cvrA,^97^ that protects genome integrity and fixes abortive DNA ligation during ribonucleotide and base excision DNA repair associated with cytotoxic adenylated DNA strand breaks. Five bound AMP molecules are shown. DNA ligase enzymes use ATP or NAD^+^ as cofactors to temporarily add an AMP group to DNA that enhances AMP ligation.^98^ Thus, DNA-adjacent ligands could facilitate DNA binding, are possibly components of a DNA ligation process, or could possibly stop the translation of the protein along DNA.

**Figure 8:**
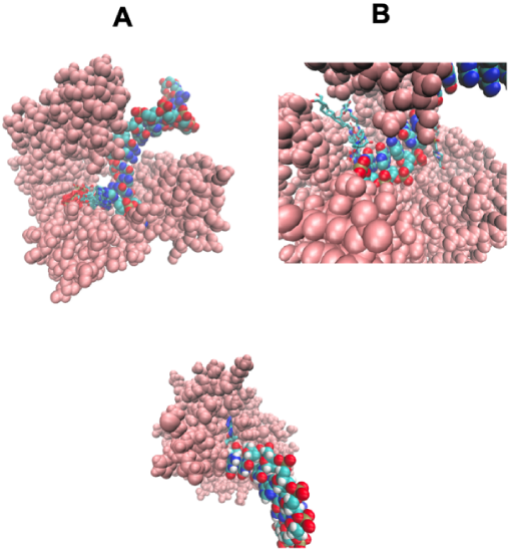
Examples how small molecules/metabolites can assist with DNA–protein binding and DNA ligation. The protein is shown in pink, and the DNA and predicted ligands from LIGMAP are shown in a licorice representation, colored by atom type, with oxygen in red, nitrogen in blue, carbon in cyan, and hydrogen in white. (A) 3khhA^95^ DNA bound to Y-family polymerase 4 whose binding is possibly assisted by 24 ligands including metabolites DCP, 2’-deoxycytidine-5′-triphosphate; DTP, 2’-deoxyadenosine 5′-triphosphate; and ACT. (B) 3t3fA,^96^ the large fragment of Taq DNA polymerase, is predicted to bind 31 ligands including DCP. (C) 6cvrA^97^ is the structure of aprataxin that protects genome integrity and fixes abortive DNA ligation arising during ribonucleotide and base excision DNA repair associated with cytotoxic adenylated DNA strand breaks. Five examples of the bound AMP are also shown and are part of the DNA ligation process.^98^

## Discussion

At first glance, metabolites and their pathways are often seen in terms of their roles in the synthesis and breakdown of small molecules, with their potential interactions with nonenzymatic proteins and DNA considered to be secondary. However, humans are estimated to contain over 100,000 distinct metabolites.^5^ Clearly, these do not play a passive role in cellular function. Indeed, as metabolomics has matured, there is growing recognition that metabolites play an essential role in biological processes^7–10^ and diseases.^14,100^ Despite this, the mechanisms by which they exert these effects is at best poorly understood and mainly phenomenological. This work aimed to clarify the general mechanistic principles by which metabolites contribute to the biochemical and physiological functions within cells.

The physiological concentration of some free metabolites is quite high,^2^ greatly exceeding the molar concentration of proteins.^1^ Consistent with the binding of multiple small molecules to proteins that are often seen in crystal structures,^27^ experimental studies^25,37^ and the fact that each protein domain has 3–5 small molecule binding sites, this study strongly indicates that a given protein is likely to simultaneously bind more than one metabolite (Table 5). Here, generalizing the idea of COLIGS, we proposed that metabolites at physiological concentrations can also remodel protein pockets and act as molecular glue. This could enable the protein to bind other metabolites at lower concentrations that would be possible in their absence due to their interaction with partner ligands in the COLIG. Given that such COLIGs are essential for enzyme catalysis, it is highly plausible that other metabolites also bind to the enzyme’s active site. These could modify enzyme kinetics by preventing product release or enhancing substrate binding as well as other types of protein function if the protein is a nonenzyme. Such interactions also occur when metabolites bind signaling proteins. Thus, within a cell, a given protein may bind multiple metabolites, challenging the idealized picture of a single protein molecule binding a single metabolite as is often seen *in vitro*. Such multicomponent binding modifies the protein’s behavior from that observed at infinite dilution.

The collective behavior of proteins, DNA, metabolites, etc., (that need not physically interact) in a living cell is often organized into pathways, a correlated set of functional events involving a collection of proteins and/or DNA (or RNA) which act coherently to contribute to cellular function. What is responsible for such organized behavior? Remarkably, we provide significant evidence that this correlated functional behavior could result from their binding of common metabolites that modulate their function either by COLIGS, allosteric transitions or metabolic entanglement of proteins that need not directly interact (whose elemental equilibria are shown in Figure 7). Such interactions could act to modify the molecular function of the constituent proteins and thereby in aggregate modify collective behavior. Of course, the particular molecular functions that are modified depend on the protein players; here, we are describing general mechanisms by which this might occur. We call such collective behavior of a given set of metabolites that could result in the creation of a biochemical pathway whose function is modified by their binding metabolites an entabolon. Even if such proteins were localized in the cell, this does not guarantee that their function is coordinated unless the proteins directly interact; yet pathways also involve nondirectly interacting proteins. Metabolites can readily diffuse in the interstitial space between proteins and thus couple their behavior even if the proteins themselves are not nearest neighbors in the cell. Using LIGMAP, in Table 3, we showed that if a pair of nonhomologous proteins (that may be multimers or monomers) bind the same metabolite, they are very likely involved in the same pathways. As further suggested by Tables 4 and 5, common metabolite binding is likely a major factor responsible for the long-range correlation of the proteins within a given pathway that gives rise to the pathway’s collective function. What is clearly required are experiments that test these ideas.

If this picture is true and metabolites play a major role in pathway creation, one might expect that ancient pathways were created by ancient metabolites, a role that they assume even today. For a representative set of nonenzymes, the top 10 metabolites ranked by the number of protein pairs that bind that metabolites in the same pathways are ancient metabolites. As shown in Table 5, these metabolites are associated with pathways that are essential for life. This is again strongly suggestive that such ancient metabolites and the resulting Entabolons are responsible for the collective protein behavior that constitutes cellular pathways.

Just as metabolite binding can act as an antagonist or agonist for protein biochemical function, metabolites might disrupt nucleic acid–protein interactions by binding within the nucleic acid–protein interface, effectively acting as antagonists. Building on this idea, we showed that metabolites are also likely to bind adjacent to the protein–nucleic acid interface. When they do so, they could stabilize protein–nucleic interactions—they could influence where transcription factors bind on DNA to initiate transcription—or they could help position substrates for DNA synthesis. Alternatively, if their binding to the pocket adjacent to a given protein–protein interface is strong, they could terminate transcription or translation.

Since most of our results are robust and invariant to details, it is likely that the conceptual framework provided here is qualitatively correct and can serve as a foundation for new research aimed at validating a metabolite-centric view of life. This is consistent with ref 30, which points out that there is evidence that the metabolism governs the biology of a cell. Thus, we agree with the view that perhaps metabolites generally regulate the biochemistry of life rather than metabolites just being regulated by the biochemistry of life. This study provides the molecular mechanisms as to how this is actually achieved. Knowledge of how metabolites create and modulate the function of individual molecules and how they influence their collective behavior has many potential applications. These range from studies on the origin of life to advancements in synthetic biology, the engineering of novel pathways, and developing more effective drug treatments in particular and therapeutic approaches in general. Thus, this work offers a comprehensive framework that further helps enable the shift from the study of individual molecules to understanding and influencing their collective behavior characteristic of living cells.

## Methods

### Metabolite Library

A library of 770 human metabolites (Table S7.LIST.metabolites) bound to PDB^27^ structures was collected. They are a quite diverse collection ranging from lipids to amino acids to essential metabolites such as ATP, ADP, AMP, or GTP.

### Construction of the Metabolite Template Pocket Library

All pockets are identified using the CAVITATOR^19^ pocket detection algorithm, which is capable of finding the pockets in which 97% ligands in the PDB are bound.^34^ Using CAVITATOR, we then constructed the template pocket library (Table S11.LIST.metabolites_pockets) of 91,269 pockets where the 770 types of human metabolites are bound.

### Overview of LIGMAP

LIGMAP, our ligand-binding prediction algorithm works as follows: Structural alignment of the entire pocket library against the largest pocket in the target protein, as identified by CAVITATOR, was done using the APoc pocket alignment algorithm.^101^ We align the entire pocket containing the metabolite against the entire pocket in the target’s structure. Empirically, we found that this provides the best precision and recall in small molecule screening.

For the assignment of metabolites to enzyme pockets, we considered a library of 29,581 enzymes (Table S2.LIST.fullset). For the pathway analysis that explores the correlation of multimeric and their corresponding pathways that bind metabolites, we considered a representative library of 32,194 metabolite pockets (Table S8. LIST.metabolites_pathway) and a representative set of 1108 target PDB structures clustered at 80% sequence identity (Table S9.LIST.proteins_pathway).

For a template metabolite to be assigned as binding to a target protein pocket, the *p*-value of the pocket alignment from Apoc must be ≤0.003,^101^ 6 residues must be identical in the pocket–pocket alignment, and at least four different ligand templates must provide a given metabolite that satisfies these criteria. These values were determined by optimizing metabolite binding in the Prot-Met data set^37^ (see Figure 2). To optimize the precision-recall values, we chose a precision of 0.66 whose recall is 0.142. Then, using the structural alignment of the template to the target pocket, we align the template ligand to the target pocket. To accommodate ligand and pocket flexibility, if more than twice the number of ligand heavy atoms overlap the target protein within 3 Å, that metabolite is rejected. In addition, if less than 3 heavy atoms are within $\sqrt{30}$ Å, the ligand is not touching the target protein; that metabolite is also rejected. The set of aligned metabolites to the target structure is provided in SI, pdb_met library. The precision of metabolite binding can be modulated by setting a threshold of the number of bound metabolites. Again, for high precision calculations, we require, for a given metabolite, that at least four templates have the predicted selected metabolites predicted to bind the given protein. If one wishes to increase the recall at the expense of precision, for the Prot-Met testing test, we can reduce the threshold of the number of template metabolites that are required to make a metabolite binding prediction from four to two. If the maximum sequence identity of the target protein to template protein containing the metabolite is <30% and if at least two metabolites of a given type are predicted to bind to the target protein’s pocket, then the average precision, recall and NPV are 0.47, 0.13 and 0.97, respectively, for 14/20 experimentally determined classes of metabolites for the Prot-Met set.

### Benchmarking of LIGMAP’s Ability to Predict the Native Pose of the Metabolite

To examine LIGMAP’s ability to predict the native pose, we examined the RMSD of the predicted metabolite’s pose relative to that of the native ligand. For a given predicted metabolite pose, we calculate the RMSD of all the heteroatoms in the metabolite. We then calculate the number of cases, *n*_<_ where the RMSD to the native metabolite pose <3 Å as well as the number of cases where it is greater, *n*_>_. The average RMSD of all poses <3 Å is collected and the average of these RMSD values over all proteins predicted to bind the metabolite is reported in column 5, Table 2. A successful prediction occurs when *n*_<_ > *n*_>;_ the ratio of the number of proteins when *n*_<_ is greater than *n*_>_ is reported in column 6, Table 2. In all cases, the average fraction of successful pose predictions is >0.86.

### Determination of COLIGs for Native Ligands

To assign a pair of ligands as being a COLIG, both ligands must have at least 5 het atoms within 4.5 Å of the protein’s Cα atoms. In addition, at least 15 pairs of het atoms in the two ligands must be within √14 Å.

### Determination of COLIGs for Predicted Binding Metabolites

We next considered the case where one predicted binding metabolite is a COLIG of another ligand bound to the native structure. To be considered, the native metabolite must have at least 10 Cα contacts with its binding protein. Then, to identify the partner metabolite, the standard LIGMAP algorithm is run with a sequence identity cutoff of 0.99, as the goal is to find examples of singly bound native ligands in the PDB. The predicted metabolite’s pose must have at least 10 contacts with the protein Cαs. We then count the number of repulsive Cα contacts defined when a ligand het atom is within 3 Å of any Cα atom. If there are more than four such contacts, the predicted metabolite is rejected. Similarly, the predicted binding pose of the metabolite cannot have more than four overlaps with the native bound ligand. To be accepted, the metabolite must also have at least 30 interactions whose distance to the native ligand >3 Å and <4.5 Å; i.e., it contacts the native ligand. If all these considerations are satisfied, the native-predicted metabolite pair is a COLIG.

### Metabolic Coupling of Proteins within Pathways

A library of 28,896 human dimeric proteins was clustered at 80% sequence identity (Table S10, LIST.dimers_human_80) giving 1106 dimer interfacial pockets. These are labeled as “inter” for the purpose of this analysis. An interfacial pocket is defined as follows: They are pockets present in the unbound monomers (see Figure 4), are absent in the dimer and which contain at least 10 buried residues in the dimer interface. Interface-adjacent pockets, termed “adj” are pocket’s that are not found in the isolated monomers, but occur adjacent to the protein–protein dimeric interface and contain at least five residues from each of the two chains.

A set of human multimeric proteins clustered at the level of 80% sequence identity was prepared, (Table S10.LIST.dimers_human_80) and contains 1,108 proteins. This set contains 440 enzymes, “Enz” and 678 non enzymes, “Non-Enz”. Each of the dimeric interfacial pockets, “inter” and interface adjacent pockets “adj” were scanned against the library of 91,260 metabolite pockets (Table S12. LIST.metabolites_pockets). For each protein, LIGMAP is run to compile a list of predicted screened metabolites, and for each screened protein, we mapped them to a set of 1822 distinct Reactome pathways^58^ (Table S12. LIST.reactome_pathways).

As shown in Figure 4, we then compare the set of metabolites associated with the “adj-vs-adj”, “inter-vs-inter,” and “adj-vs-inter” equilibria. For a given protein pair and a given type of metabolite, we determine the number of cases where they are found in the same Reactome pathway. Here, for each pair of proteins, we exclude all cases where any one of the monomers in the dimer has >40% sequence identity to another monomer in the other dimer. This ensures that we are not considering the trivial case of highly homologous proteins binding the same metabolite in the same pathway.

For the given pair of proteins that bind the same metabolite, we report the number of common pathways that the pair participates in, the total number of possible pathways (1823) and the number of pathways each protein participates in. We then calculate the probability that the number of shared pathways is random as determined using the hypergeometric distribution.^102^ Here, we are implicitly exploring the idea that within a given pathway metabolites implicitly couple the function of two dimeric proteins and/or their monomers by modifying their state of association and thereby function.^103^ This is a test of the metabolic entanglement idea.^34^

### Mapping of Proteins Binding Common Metabolites to Their Pathways

For each protein in a given pathway, LIGMAP is used to predict which human metabolites, if any, it is likely to bind. Two proteins have a metabolic contact if they bind the same metabolite. We first consider the largest set of proteins that share a common binding metabolite.^104^ We then extend this by considering proteins that bind to the metabolite in the original set but also bind a different common metabolite with an original member protein. We then join all these proteins that bind the second metabolite to the aligned pathway. The process is iterated until convergence. The number of metabolites is just the number of such pairs of proteins that bind the given metabolite in the resulting subpathway. The number and fraction of proteins in the given pathway that are members of the strongly connected component along with the up to top 5 binding metabolites are reported in Table 4 for entangled proteins and in Table 5 for human, monomeric non-enzymes.

### Binding of Metabolites to Proteins Involved in DNA–protein Interactions

To identify metabolites that likely disrupt protein–DNA binding, for a given PDB structure that contains a protein bound to DNA, we compiled a list of proteins that are at least 50% identical to the target protein but which do not have bound DNA bound. To be considered a candidate for subsequent analysis the RMSD of the target and template homologous proteins must be <2 Å. The superimposed ligands of the template protein onto the target protein must have at least five heavy atom contacts (<4.5 Å) with the target protein. For a ligand to be considered to bind adjacent to the DNA–protein interface, it must have at least 5 heavy atom contacts with the protein and for DNA binding, at least 10 heavy atoms located between 3 Å and 4.5 Å of any DNA heavy atom.

## Supplementary Material



## Notes

Table S1.LIST.pdb_protmet_unique.txt: list of proteins in the Prot-Met set; TableS2.LIST.fullset.txt: list of representative enzymes screened against human metabolites; Table S3.LIST.protmet_MET.txt: list of metabolites in the Prot-Met Set; Table S4.LIST.enzymes_aln_MET_het.txt: list 1112 human enzymatic proteins used to evaluate the performance of LIGMAP; Table S5.LIST.enzymes_colig: list of 2233 enzymes that have predicted COLIGS; Table S6.LIST.nonenzymes_human.txt: list of 4479 human protein monomers that are nonenzymes; Table S7.LIST.metabolites.txt: list of 770 human metabolites used in pathway screening; Table S8: LIST.metabolites_pathway.txt: representative library of 32,194 metabolite pockets; Table S9.LIST.proteins_pathway: representative set of 1108 target PDB structures clustered at 80% sequence used to screen multimeric proteins; Table S10.LIST.dimers_human_80.txt. list of 28,896 human dimers used to examine metabolic coupling within protein pathways; Table S11.LIST.metabolites_pockets.txt: library of 91,269 protein pockets where the 770 human metabolites are bound; Table S12: List.reactome_pathways.txt: list of 1822 Reactomes used in the multimeric protein pathway analysis; Protmet_pdb: predicted poses of the bound metabolites in the Prot-Met protein set; Pdb_met library: predicted poses of the bound metabolites in the LIST.proteins_pathway set; Ligmap.f: source code of LIGMAP (ZIP)
